# An economic model of the drives from Friston’s free energy perspective

**DOI:** 10.3389/fnhum.2022.955903

**Published:** 2022-10-20

**Authors:** Gustaw Sikora

**Affiliations:** British Psychoanalytical Society, Institute of Psychoanalysis, London, United Kingdom

**Keywords:** economic model, theory of drives, free energy, somato-psychic frontier, psychoanalysis

## Abstract

This paper is focused on the theory of drives, particularly on its economic model, which was an integral part of Freud’s original formulation. Freud was aiming to make a link between the psychic energy of drives and the biophysical rules of nature. However, he was not able to develop this model into a comprehensive system linking the body and the mind. The further development of psychoanalytic theory, in various attempts to comprehend the theory of drives, can be described as taking different approaches. Some of them equate drives with bodily impulses, others abandon the economic model, a few stay with Freud’s original model. I believe that the Friston notion of free energy and the hierarchical model of the brain allows us to develop this model and to integrate the economic model into some contemporary theories of drives. I argue against those theories equating drives with biological impulses. My arguments are supported by Freud’s project itself but also by recent developments within neuro-psychoanalysis describing the process of mentalizing homeostasis, interoceptive signals and relations with caregivers. I argue for those theories which see the drives as psychic forces, which through developmental processes and cathexes acquire aims and objects, and become intertwined with impulses originating internally and externally, such as affect, interoceptive impulses, perception of the external world, and impulses from erotogenic zones in particular. Here, I present my analysis of the compatibility and consistency of free energy and the hierarchical model perspective, and two psychoanalytical traditions of thoughts: French psychoanalysis and the post-Kleinian school of British psychoanalysis. In particular, my analysis focuses on the contemporary Kleinian notion of unconscious phantasies, especially Bronstein’s description of their semiotic aspects. Secondly, I look at Segal’s view of drives as dialectic forces of adaptation vs. conservatism. Analyzing the French tradition, I focus on Green’s perspective on the drives, Lacan’s distinction between drives and desire, and Penot’s description of the process of subjectivation. I conclude that free energy, as described by Friston, can be seen as a source of the drives’ energy and the process of minimizing it is an equivalent of what Freud described as binding the free energy, in which psychic unbound energy acquires distinctive features and becomes bound.

## Introduction

Psychoanalytic metapsychology is an attempt to build a coherent model of the mind. Although in this endeavor we make use of data from clinical observations and research, many parts of emerging models remain speculative (see Britton, 2015). In recent years, there have been many attempts to use discoveries from natural sciences to enhance and clarify psychoanalytic knowledge.

This paper focuses on a particular aspect of one of the psychoanalytic models, namely on the economic aspect of the theory of drives. It is based on assumptions which Freud made when he formulated his model. For clarity, in this paper I use the term “psychoanalysis” to describe models of the mind which use the theory of drives.

The theory of drives is one of the fundamental pillars of psychoanalysis. For Freud, the economic model was an integral part of this theory, which aimed to make the link between psychic energy and the biophysical rules of nature (Freud, 1915a). However, he was unable to develop this model into a comprehensive system linking body and mind. That has left psychoanalysis with many questions, one of them being: what is the nature of the energy of the drives? One might also consider this question from the perspective of the twofold nature of the phenomenon: the energy and the content of the drives (quantity and quality) and focusing on a qualitative aspect only. However, nature of energy still remains an open question.

In the various attempts to comprehend drive theory, most of the further developments of psychoanalytic theory can be described as following two different avenues. One equates drives with recognizable bodily impulses; for example, Sandler and Schachter (2014), Solms (2018). The other ones follow Freud (1933) in his claim that drives are “mythical entities” consisting of pure psychic energy; but they more or less abandon his economic model (see Bell, 2015; Penot, 2017). There are exceptions to these: most notable Green, 2010, 2015a,b, who follows Freud’s economic model without explaining the biological sources of the drives’ energy but not denying them either. I will not consider in this paper those models that abandon drives completely, as abandoning drives means a complete departure from the psychoanalytic model as it is understood in this paper.

This paper argues that Friston’s notion of free energy and the model of the brain as a “probability machine” (Friston and Stephan, 2007; Friston, 2010) provide us with knowledge that allows us to integrate the economic model into the contemporary theory of drives, including object-relation theories. Using developments within neuro-psychoanalysis that describe the process of mentalizing homeostasis and interoceptive signals, this paper attempts to develop a hypothesis concerning the nature of a particular function of brain/mind which helps the free energy of the drives to become bound, and the drives to acquire content.

The paper also tries to match this hypothesis with those theories that see drives as psychic forces which, through developmental processes, become intertwined with impulses originating internally and externally, such as affect, interoceptive impulses, perception of the external world, and impulses from erotogenic zones in particular.

While seeking for compatibility and consistency of free energy, and a hierarchical predictive model perspective with traditions of psychoanalytical thought, I will use mostly French psychoanalysis and the post-Kleinian school, as I find that both are strongly rooted in Freud’s original formulation of the drives. This analysis is mostly focused on the contemporary Kleinian notion of unconscious phantasies as representations of the drives, in particular Bronstein’s (2015) description which uses Kristeva’s work on semiotics (Kristeva, 1984, 2000) and on Green’s (2015a) understanding of drives as forces of binding and unbinding (see also Penot, 2017).

There have already been many attempts to bridge neuroscience and psychoanalysis, all facing difficulties which come up from the complexities of both fields, different epistemologies, terminologies (they both use similar or the same terms to describe different phenomena), and many more. These often lead to misunderstanding and sometimes premature rejection of such attempts by psychoanalysts. On the other hand, they sometimes lead to the building of radical reformulations of psychoanalytic theory which are not compatible with clinical observations and practice. To minimize these problems, I will clarify meanings of analytic terms I am using as much as possible, yet being aware that some analysts use those terms differently. Also, despite my starting point being Freud’s formulation, I am focused on compatibility with contemporary psychoanalytic knowledge.

Ultimately, my analysis concludes that free energy, as described by Friston and Stephan (2007), Friston (2010), can be seen as a biophysical source of the drives, and that the process of minimizing this free energy is equivalent to what Freud described as a process of binding energy, in which purely unbound energy acquires distinctive features. It offers an answer to the question of what force is behind crucial processes of cathexis/decathexis. The paper concludes by drawing attention to some possible implications of this hypothesis for psychoanalytic metapsychology.

## Theory of the drives

First, I need to seek as precise of the term drive as possible which Freud used in his model. There is a certain confusion about this term, especially in English speaking literature, mostly due to Strachey’s translation of Freud (Strachey’s, 1957 [in introduction in Freud, 1915b], pp. 111–116). Significance of differentiation of a drive and an instinct was earlier elaborated by Laplanche and Pontalis (1973b). Conrad (2021), to whose work I am indebted, believes that “the drives no longer occupy a preeminent position in the psychoanalytic theoretical landscape” because many analysts think that they are “simultaneously too abstract and too simple to be a serviceable concept.” Although, this may be true for some schools of psychoanalysis, there are also prominent analysts who have been of the opposite view, among them Segal (1985), Feldman (2000), Green (2015a), Bell (2017).

I start from the use of the term “drive” (original German term Trieb), prior to Freud’s writing. Conrad (2021, pp. 493–494) claims that a crucial figure in developing the meaning of this term was J. F. Blumenbach, who introduced the term “Bildungstrieb” (drive of development) to describe a force which pushes a living organism toward a determined shape; however, he saw it as “a mysterious force that could be clearly identified, while its cause remained unknown” (ibidem). I would add here that in contemporary language, “toward its determined shape” could be formulated as “toward organized structure.” Furthermore, drive (Blumenbach’s Trieb) can be seen as “anti-entropy” force, if we put it (drive) in the framework where entropy represents chaos and is the opposite of organized structure.

Further XIX century considerations on motivation brought about a better understanding of the complexity of it, including the works of Spencer (1870), Schneider (1880). Conrad (2021) summarizes it as follows: “a spectrum from animal to human as the progressive increase in complexity of motivation, with the simplest animal organisms experiencing feelings purely mechanistically, more complex animals being motivated perceptually, still more complex animals being motivated by representations, and finally the most complex animals (i.e., humans) being motivated by thoughts” (ibidem, p. 496). At the same time, Schopenhauer (1958) made instinct central to his understanding of human will. He believed that the ultimate force behind the complex phenomenology of human behavior is instinct, which is a purely biological force and is always hidden in various behaviors and actions, but the goal of it is always the same. Finally, we can look at Nietzsche’s (1881) work where he tried to elucidate the role of drives. He emphasized that the same situation (stimulus) can evoke very different responses from different individuals, depending on which drive is active at the very moment, emphasizing that different behavioral outcomes always aim to satisfy the drive involved. In other words, humans are driven by the same stimulus to satisfy different outcomes determined by the qualitative features of the drives involved.

I believe that Schopenhauer’s and Nietzsche’s takes on this problem elucidate a difference between an instinct and a drive in German speaking world of the time.

All of this was surely known to Freud when he started his own work on the formulation of drives. His scientific ambitions did not allow him to be satisfied with philosophical speculation; he wanted his theory to be proved by natural observations or other natural sciences (for more see Jones, 1953). Nevertheless, Freud’s writings on drives leave considerable room for interpretation in attempts to define them. His own struggle with how to be precise in conceptualizing them can be seen in his words when, on the one hand, he described the theory of the drives as a fundamental concept [*grundbegriff*] (Freud, 1915b), while on the other he called them “mythical entities” (Freud, 1933). However, he drew clear lines that constitute his understanding of the drives and their central role in a psychoanalytic model of the mind. He elaborated on the qualitative dimension of them but in regard to their source, [Quelle] he was realistic about the limitations of his knowledge; he wrote: “The study of the sources of instincts (drives) lies outside the scope of psychology…in mental life we know them only by their aims” (Freud, 1915b).

I’ll summarize how Freud described the drives. Firstly, drives [*trieb*] are psychic forces that are distinguished from instincts. The latter are more biological in their nature, more automatic, and are less prone to modification or alteration. Drives are modified during development. Drives are described by four features: source, aim, object and pressure (Laplanche and Pontalis, 1973a,b). Aims and objects are changing and the outcome of such development varies (Freud, 1905). [for more arguments for differentiating instinct and drive see Conrad, 2021, p. 499–503].

Secondly, source and pressure, which also describe drives, remain the same (Freud, 1915b). The latter is conceived as a quantitative factor of the drives’ energy.

Thirdly, the source of the drives, identified as the Id, is “on the frontier between the mental and the physical” (Freud, 1905, 1915b). According to Freud, aim and object are attached by this energy and subsequently we have the “vicissitude” of the drive [*triebschicksal*], which is an entirely psychic entity. The nature of this energy and in what way it is attached to the aim and object remained unexplained. The part of the vicissitudes which bears energy, or in other words operates as a vehicle for already bound energy, is affect. Affects themselves have energy but the energy in question is one which links aim, object and affect in a one entity.

Fourthly, the drives have a dualistic nature (Freud, 1920); that dualism is the regulating force of the representatives (aims, objects) of the drives, which determines the drives’ vicissitudes (see also Bion, 1963).

Therefore, we can describe drive as a demand on the mind; that demand starts with stimuli and binds certain ideas with certain objects which become salient to the individual. Also, it is bound with the “quota of affect,” which determines the strength of the drive. This constitutes the qualitative features of the drives and gives them pressure coming from energetic aspect of affect. For Freud, there are two crucial elements of the drive: affects and ideas. Ideas describe aim and object, and affects are transpositions of the quantity of energy (Freud, 1915a,c; see also Laplanche and Pontalis, 1973a). It is very important to note that affects are not the same as drives. They may be bound with ideas or split off from them. They bear a quantitative dimension of energy [*affektbetrag*], but they are part of the vicissitudes, not the source of the drives’ initial energy (Freud, 1915a). Affects are basically conscious and subjective; Freud tried to make “affects” distinct from “quotas of affects,” which more closely correspond to drives but are still not the same. “Quota” reflects the fact that affect’s energy must also be bound (cathected), however, cathected is also strength of affect and that which bears the quantitative part of a drive and constitutes a pressure. Together with ideas (qualitative part) they constitute the vicissitudes of the drives. Conrad (2021) writes “[drive]…arouses an affective state that serves to evaluate the world in such a way that makes the salience of the object justified” and I would add to make object “desired.” Therefore, we could say that the crucial process in forming drive’s “content;” that is: aim, object and accompanying affect, is cathexis (Besetzung). Freud has never given a rigorous definition of this term; however, it is widely understood that it is a process which creates intentionality and a value object (Laplanche and Pontalis, 1973a). The opposite process of decathexis must also be active in the human mind so as to allow changes in the drive’s aim and object. Again, that leaves us with the question: what force/s are behind cathexis and decathexis.

My interpretation of Freud’s claim that “drives are mythical entities” is that we can use the psychoanalytic method to research qualitative features of the drives, which are attached to their energy and can be modified; but we have no access to the drives’ sources of energy as such because they are “pure” energy. In the clinical sense, it means that psychoanalytic work with the unconscious is dealing with the drives’ unconscious derivatives. Freud wrote “I am in fact of the opinion that antithesis of conscious and unconscious is not applicable to drives. Drive can never become an object of consciousness-only; the idea that represents it can. Even in the unconscious, moreover, a drive cannot be represented other than by an idea. If the drive did not attach itself to an idea, or manifest itself as an affective state, we could know nothing about it” (Freud, 1915a). Regarding the source, Freud postulated “continuously flowing source of stimulation,” (Freud, 1905) or in other words a permanent demand on the mind coming from unknown source. I would suggest that Freud is referring here to source as continuing process which “produce” energy which demands [compare with definition of source (The Cambridge English Dictionary, 2022)].

The fact worthy of noting is that the number of drives is not limited and they can be shaped in a variety of ways. Making a list of them, or classifying them in nozological manner, seems to be impossible in this system. Therefore, Freud after a few attempts finally made a simple distinction between Life drives and Death (Anti-Life) drives (plural and various in their expressions). This distinction seems to be based on a practical/clinical criterion, which is the dimension: promoting development vs. restraining development (of the mind).

The further development of psychoanalysis had to deal with those considerable, visible gaps in the theory of the drives. A complete analysis of that development is beyond my scope and is not the purpose of this paper. Also, such an analysis would be almost impossible because, as Sandler and Schachter (2014) argue, “every analyst has private theories and is influenced by different schools.” Therefore, I outline only certain tendencies in that development; this allows me to explain my choices of models for further argument. Later, I focus on selected propositions which, in my view, have the theory of drives based on Freud’s model described above; and also provide knowledge which is to some extent compatible with the free energy model. I am fully aware that not all analysts will agree with my outline and selection. Also, as it is only an outline, my description is simplified and does not give justice to all analytic thinkers. Most importantly, my selection here is dictated by the purpose of this paper, which is not to compare and analyze psychoanalytic schools of thoughts, but to develop a hypothesis that answers the question: What is the primal energy of the drives?

One line of that development was to “unhook the concept from the biological theorizing that formed part of Freud’s initial presentation” (Bell, 2015). Bell is writing here about the death drive, but I believe he means the whole of the drive theory.

One of the proponents of this approach is the Kleinian school, which has developed a complex theory of the drives but has effectively abandoned the biological aspect and the economic model of the drives. The very question of the drives’ origins is often replaced by the claim of innate purposive drives. Klein herself tended “to assert complementarity between her emerging viewpoint and Freud’s theories” (Klein, 1940), but many of her followers believe that her work proved that the economic model was wrong. Spillius et al. (2011a) argue that Klein’s understanding of the work of mourning in the context of infantile conflict of the depressive position “has more bite and also more depth than Freud’s economic formulation of mourning, and incidentally shows the limitations of the economic model.” However, showing limitations is not equal to disproving a claim. It seems to me that Klein has presented a greater complexity of the dynamics of mourning; however, this does not disprove the economic model but only gives it a new dimension. In a critique of Klein’s work, York (1971) claimed that she exchanged the quantitative model of libido for the quantitative balance between the life and death drives. I would suggest that although she was not interested in the quantitative aspect of the economic model, she drew attention to the issue of balance between the drives in the dualistic model. I fully agree with O’Shaughnessy (1981) that “Klein described something similar to the pleasure principle but from another perspective… This is an object-relations perspective on the discharge of displeasurable tensions and stimuli.” In my view, the above description of the “content” of the drives justifies thinking about drives from an object-relation perspective, or even more - the understanding of stable patterns of object-relation demands, situating them in the frame of theory of drives. However, it is widely understood that most Kleinians are not interested in the biological source of the drives; what is understood by many psychoanalysts is that the drives in Kleinian metapsychology are metaphors. I believe this is over interpretation and that the Kleinian approach does not exclude the biological source of the drives.

Furthermore, biology is abandoned in the approach represented by the Lacanian tradition. For Lacan (Lacan, 1977a, pp. 235–243), the distinction between instincts and drives is crucial and the drives are completely removed from biology. In his complex elaboration, he distinguishes between need, desire, demand, and drive (Lacan, 1977a, p. 162). Drives in his theorization are purely mental entities; thus, the energy of drives seems to be truly metaphorical. Noteworthy also is his understanding of the dualism of the drives; Lacan (1988) does not differentiate death and life drives but sees destructiveness in the excessiveness and repetitiveness of drives. It accepts the economic dimension, but it does not seem possible to link this model with the original idea of drives as emerging on the “somato-psychic frontier.”

A different and interesting proposition is presented by Green (2015b). He adheres to Freud’s original idea that drives have to be considered in a three-dimensional context: economic, dynamic, and topographical. Simplifying, it leads him to an understanding of the drives as forces of binding and unbinding; drives are the “powerhouse” of the development of representations linked with the quota of affect; or in other words, drives make a “link between psychic activity and the body” (Green, 1997). Penot (2017), who shares similar views, observes: “I would say that no drive trajectory can be accomplished with binding libido alone, and without benefiting from the *dissociative* force necessary for the dynamic of *organization*−*disorganization* that underlies the dynamic destiny of every drive pair.” I cannot do justice to this complex theorization but only want to underline a few important points for this paper. Firstly, Green suggests that drive energy is bound into representations and quotas of affects that constitute cathected objects (including a relation to an object). Secondly, such objects are not only imaginary or real persons, but also ideas. Thirdly, he sees the energy of the death drive as a force of dis-objectification/unbinding. Fourthly, he sees these two forces as oscillating throughout life (Green, 1997). Again similarly, Penot (2017) claims that without unbinding/de-cathexis, subjective life would not be possible. His point is that the development of the psyche needs both drives to balance the forces of binding and unbinding, which provides a balance between the stability of cathexis and flexibility, i.e., providing room for change. One can say there is some similarity between this approach and what was said above about Klein and her thinking about a balance between the life and death drives.

A very different view of the economic model is presented by some members of the contemporary Freudian tradition, although many contemporary Freudians do not fully share this presented view. Regarding the role and source of the drives, Sandler and Schachter (2014) claim: “These early experiences are essentially bodily: ‘The ego is first and foremost a bodily ego’ (Freud, 1923), derived from multiple physical contacts with the mother/caretaker, and in response to the bodily pressures of the drives (hunger, thirst, cold, pain, etc.).” In this interpretation, the drives are biological impulses (like hunger), and their energy is later represented by affect. This development is described as follows: “The role of the body in its physical reality and its representation within the mind is fundamental in understanding the earliest affective experiences. The body is the seat of the drives and therefore the site of the earliest affective experiences, which over time become represented at a psychic level as the ego, and its functions develop within the matrix of the relationship with the mother” (Sandler and Schachter, 2014). In this approach, in contrast to Lacan and Klein, the emphasis is on biology and affect but drives become equated with biological needs, which, in my view, radically changes the notion of the nature and the role of drives. Although I agree that bodily impulses such as hunger, cold, and particularly impulses arising in erotogenic zones, are included in representations of drives through cathexis and constitute a very important part of these representations, I argue for the model in which the drives (in their economic sense) are forces behind cathexis, and that they are very different entities from needs. In fact, without this distinction we would not need the separate concept of the drives.

Some middle ground in this dilemma is presented by the Paris School of Psychosomatics. Aisenstein and Smadja (2010) describe their approach as follows: “In the theoretical model of the Paris school, instinctual drives have their source in bodily excitation. Their role is to deal with the tension thus created. If the sum of excitations continues to be excessive, the functional systems become disorganized and the mental apparatus overloaded.” This theoretical framework uses economic terms, considering different ways of discharging excitations and the different obstacles encountered on the way. In Green’s (2010) discussion of the Paris school, he suggests that their theory of drives is too biological and raises a question about the dualistic nature of the drives. However, it is possible to interpret this approach differently; in fact, in a way that is similar to Green, by taking excitation as an energy that is different from bodily needs, leaving the nature of the energy (or excitation) unexplained.

Interestingly, Aisenstein and Smadja (2010) tried to address criticism of the abandonment of the dualism of the drives by introducing Ameisen’s discovery of apoptosis as a proof of the death drive and incorporating it into their theorizing on the drives. Leaving apart the fact that apoptosis is a regulatory mechanism within a body and not a self-destructive activity, their claim makes Green’s (2010) suggestion of over-biologisation somehow more valid.

However, the original Fain and Marty’s (founders of the Paris School of Psychosomatics) model is explained by Fain (2018) as follows: the life drive is excitation within the body and he believes that the nature of the death drive lies in passivity; it is fending off impulses and affect. He sees this pair of opposite drives as an important regulatory mechanism and proposed different names for them: Eros and Anteros (Fain and Braunschweig, 1971). This is a similar approach to one presented by Penot (2017) in which the opposition of drives serves a balanced development. Marty (1976) describes the development of what he calls organization (of drives) as follows: “each level of organization, the new functional ensembles, include a certain number of pre-existing, as it were constitutive functions, at the same time as a new evolving ensemble gives the functions that constitute it a new form of life;…Thus, the new evolving ensemble seems to only leave in place a kind of management which takes on a hierarchical role of great importance.” It seems to explain how representations of drives evolve and suggests their multi-layered hierarchical structure, but again leaves the question of the forces which cause those changes (the energy of the drives) unanswered. The location of this model in terms of a constantly hierarchically rebuilding organization of drives is similar to that of the Kleinian model, but it explicitly includes the economic dimension of the drives.

Further along the way toward a biologisation of the drives, we can encounter the work of Solms (2018), Solms and Panksepp (2012), Panksepp (1992). They see affects as providers of energy for developing the mind and bodily, emotional, and sensory needs, which correspond roughly to the current usage of the terms “drive,” “instinct,” and “reflex.” (Solms, 2018). This effectively equates needs felt as affects with drives, and erases the distinction between instincts, needs and drives. Although undeniably affects bear energy which constitute pressure, that energy cannot explain how it is linked with aim and object in relatively stable entity. Further, it led Solms (2021) to claim that the “drives are conscious and are in fact the source of all consciousness.” This brings an interesting but controversial spatial dimension to the theory of drives, and also makes clear sense in regard to economy. However, as we can see in Solms’ above claim and in his idea of the “Conscious Id” (Solms, 2013), he effectively departs from the model described by Freud in which drives are separate entities from needs and affects, and are never conscious (see quotations above). In his model drives have a radically different meaning, so I would prefer to consider it to be an alternative proposition to Freud’s model rather than revision of his (Freud’s) model. If we attempted to locate Solms’ model in Freud’s, we would say that the concept (traditional) of drives is unnecessary, and could be replaced with the quota of affect but again it doesn’t explain in what way affect energy is attached to specific aim and object of drives. On the other hand, if we introduced drives as a separate entity to this model, we would see Solms’ “Conscious Id” rather as an affective part of the Freudian bodily ego. In my view, this approach develops important aspects of the work of the mind, drawing on and sometimes necessarily modifying psychoanalytic knowledge; for example it would add to Contemporary Freudian (Sandler and Schachter, 2014) list of needs relational needs. However, as regards the theory of the drives, they abandon the key assumption that drives are energies on the frontiers of soma and psyche, distinct from needs and affect; in other words, drives provide energy (real, not metaphorical) to the mental apparatus, but impulses (both external and internal), including needs, are dealt with by the mental apparatus and gradually become its content. Solms’ idea seems to be an alternative to contemporary psychoanalysis rather than an alteration which could be included in it.

In conclusion to the above overview, I am inclined toward those models which use the notion of drives as distinct from needs and affects, but also leave room for the economic dimension. From the clinical experience perspective, these models represent well what we see in consulting rooms: a variety of configurations of drives [It is also Freud’s view: “No objection can be made to anyone’s employing the concept of a drive of play or of destruction or of gregariousness when the subject-matter demands it” (Freud, 1915b)], plasticity of drives observed in analytic process and normal development alike. Equally, the economic dimension seems to me to be necessary to explain the forces of cathexis and decathexis; this is also clinically important for understanding common clinical facts like “compulsion to repeat,” transference or the process of “working through.” An additional argument against a unified list of drives is Fenichel (1935), warning against a simplistic take on the death drive (I believe it can be seen in the light of all notions of purposeful unchangeable types of drives), which can replace the analytic attitude of exploration with deterministic thinking.

In my proposition, I will use mostly the Kleinian model of unconscious phantasies and the Green model of drives. The latter is theoretically close to the Paris School of Psychosomatics and one can see also parallels in my proposition to this model. In some elements, we can also find compatibility with Lacan’s work.

## Models which can be compatible with the economic model

Here, I look closer at those approaches which distinguish drives from needs and affects, and can support the model I argue for. I present only elements of these complex theories, aware that I am omitting many crucial parts of these theories and the differences between them. However, these selected elements are important for this paper’s objective as they help to address a gap in the economic aspect of the drives in the presented model.

I shall start with the Kleinian notion of unconscious phantasy from the best-known definition of unconscious phantasy: “the ‘mental expression’ of instinct (*drive in the context of this paper^1^* “) is unconscious phantasy. Phantasy is in the first instance the mental collar, the psychic representative of instinct (*drive*). There is no impulse, no instinctual urge or response, which is not experienced as unconscious phantasy” (Isaacs, 1948). Later, Isaacs (ibidem) writes: “In early life, there is indeed a wealth of unconscious phantasies which take specific form in conjunction with the cathexis of particular bodily zones.” Isaacs also says that unconscious phantasy is “the primary content of unconscious mental processes”. My understanding of these claims is as follows: drives (as energy) are forces behind a building up of the content of the Unconscious, incorporating all what is experienced by a human, starting from bodily experiences (including affect which is perceived as bodily experience). Later, it includes all impulses, defenses, perceptions, and activities (Segal, 1964, 1994; Spillius et al., 2011b). Blass (2017) claims: “phantasies aren’t conceived of as [only] the content of the mind but rather as the material itself of which the mind is made up of.” This can be interpreted as the mind is built of elements which each contain a particular representation of momentary experience (this includes self, object, stimuli felt at the moment, need, way of satisfying need, affect etc.) perception of subsequent moments is possible only through lenses of existing phantasies; in effect, new experiences are building up newer phantasies “above” prior ones, but prior ones are part of the building material. This creates a kind of hierarchical structure where the earliest phantasies influence later ones, but new phantasies modify this entire structure by implementing new elements (of course, in some pathological states we can see that such modifications don’t take place and the individual affected is living in early primitive phantasies). In this interpretation, the unconscious phantasies are organized representations (in the Freudian sense) and affects; the latter can be understood as the vehicle for energy. However, most Kleinian writers do not pay attention to the energy dimension, but it is an important question about what forces attach different elements of experience in the organized entity of phantasy (pattern, representation) and what dynamic is involved, according to the fact that phantasies may be or may not be modified. In my view, it is a question of the nature of cathexis and decathexis. Some post-Kleinians theorize on that question, assuming that the drives have an innate purpose. I cannot find supporting evidence for this, except that primal needs and biological stimuli are obviously parts of any phantasy. However, observable and experienced purpose seem to be acquired in the process of development.

Bronstein (2015) gave her account of unconscious phantasies, elaborating on the early stages of constructing phantasies. She suggests that the most rudimentary experiences like sound, rhythm, or indeed interoceptive stimuli, are included in forming phantasy; thus, they become meaningful even before they constitute representations (in Freudian sense). My understanding of what “meaningful” means in this context would be that those experiences, through a process of cathexis, become linked with affects. Thus, they are affectively meaningful or, in other words, underpinned with energy through cathected affect. As Solms and Panksepp (2012) have shown, needs and affects are purposeful; we can speculate that that might be the mechanism through which drives as energy acquire purpose in early stages of their development.

Bronstein (ibidem) also argues that “even though early unconscious phantasies might be modified, organized and structured in a symbolic way to form the latent content of what will become manifested as part of a coherent integrated discourse, the form which they adopt is often imbued with raw emotional components, a contribution from the paranoid-schizoid position and from primitive phantasies.” I would suggest that they are always to some extent present even if this cannot always be observed. I can support my view by Britton’s (1998a,b), Vermote’s (2014) work on Bion’s (1965) notion of transformation. They both, in different ways, argue that the development of the content of the Unconscious is a dynamic movement up and down, creating multi-dimensional space where earlier and later introjected elements influence each other. My understanding of Bronstein’s contribution is that phantasies (as the structure) can be modified by new incoming experiences but have a multi-layered structure, from primitive to well-organized symbolic substructures. They are organized hierarchically, the latest and most advanced having the biggest impact on conscious mental life. However, these layers are in constant interaction and influence each other in two ways: top-down and bottom-up. Here, I see similarities to the Paris School of Psychosomatics’ notion on the organization and development of drives described above (Marty, 1976).

Segal (1994) sees phantasy as potentially destructive and constructive at one and the same time. She believes that phantasy has the capacity to impose on reality, but also that phantasies are hypotheses for testing reality. In the context of phantasies as representations of drives, she sees duality of drives as forces of conservatism vs. adaptation. In my view, the former may be seen as a stabilizing factor of cathexis but also as an important factor in repetition compulsion (constructive and destructive respectively). The latter may be seen as a capacity to predict and a factor in the development of thinking, but a potential cause of instability, confusion and chaos if it prevails over stable cathexis (again constructive or destructive force).

This notion of unconscious phantasies provides us with a description of the content of the Unconscious and the mechanisms by which this content is built; it also describes the roles and functions that phantasy plays in mental life. Also, it can be seen as another perspective on the place of drives in the conflict between the pleasure principle (immediacy of satisfaction without a need for adaptation) and the reality principle (search for new ways for achieving satisfaction). I find here similarities between Segal’s notion on destructive vs constructive forces and Lacan’s (1977b, 1988) understanding of the Life/Death drive dilemma; both seem to see it as rather a function borne from the intensity of the forces behind drives in the moment, than the purposeful aim included in its content.

It is worth noting that this notion of the relationship between the content of the Unconscious and conscious processes is in line with what Hopkins (2016) describes as virtual reality and the mediating role of conscious processes. My understanding of his notion is that a virtual reality is a space where phantasy/hypothesis and actual realm are negotiated [compare with Segal’s (1952) writing on function of dream and imagination].

However, the Kleinian model doesn’t answer our important question: what energy is propelling this process? Like Freud (1909), Klein (1927) answered these questions similarly: these processes and primal phantasies are innate and phylogenetically inherited. Followers like Money-Kyrle (1956), Bion (1962), who used the word “pre-conception” to describe it, developed ideas about the content of the Unconscious at the beginning of human life, but could not locate its source and stayed with “innate.” It is very likely that a human is born with some proto-phantasies, but this doesn’t explain further development, and questions about forces propelling this process remained unanswered.

Seeking for a contemporary model of drives which is compatible with free energy notion also, French psychoanalytic tradition offers a very interesting take on the drives, which seems to be the most in line with Freud’s original formulation. I will use here mostly A. Green’s body of work. Although André Green’s (2010, 2015a) theory of the drives is significantly different from the Kleinian interpretation in terms of the structure of “content” of the drives, I do not find them contradictory.

In his theorization on the Unconscious, Green saw the representations of self, objects, and ideas as separate entities from affects. However, he claimed that they interact, and that affect plays a role as a vehicle for energy; I assume here that affect energy constitute pressure but drive energy link aim, object and affect. He emphasizes the dynamics of this structure with movements between different layers of the mind. I find particularly important his understanding of the drives as forces of binding and unbinding (investment and disinvestment, cathexis/decathexis). This could be seen as a universal model of the economic and dynamic dimensions of the fate of drives which, in my view, can be applied to the Kleinian model of unconscious phantasy as representatives of drives and their cathexes, which could be understood as a description of the content of the Unconscious. Here, it is important to notice nuanced differences in the use of the terms: representative, representations, in both bodies of work. Green (2015b) writes about three meanings of it, see below; Kleinians have a less refined definition of this term, compared with the above use of it by Issacs and Bronstein. Simplifying, Green’s theory of the transformation of the drives can be described as follows:

First movement: “endo-somatic” source of energy (unknown) ⇔ somato-psychic frontier (drives as energy) = representative of drives.

Second movement: Drives (somato-psychic frontier)⇔ Unconscious = representations (ideational) + quota of affects.

Third movement: Unconscious ⇔ Pre-conscious = representation (word) + affects.

Fourth movement: Pre-conscious ⇔ Reality = perception + action.

If we assume that the ideational representation contains a salient object and aim, we can find this model very similar to Nietzsche’s description of drive (described above).

All elements of this system can and do influence each other in top-down and bottom-up directions, and also interact within the same “layer.” For example, action has an impact on perception and vice versa; similarly, affect and representation. Also, perception has an impact on representation, and vice versa. This includes all directions of influence. Cathexes are guarantors of the stability of the structure. Changes to the structure demand de-cathexis. Forces behind this dynamic structuring are the drives: the life drive as a force of binding and the death drive as unbinding. Green claims that binding applies not only to object-things but also to ideational representatives. Altogether, Green’s model builds a complex, multi-dimensional structure of the mind. Penot (2017), who also represents the French tradition, adds to that an important observation. He claims that only the coexistence of two drives (the forces of cathecting and de-cathecting) can promote development. Without de-cathexis, there will be stagnation and no development will be possible. Without cathexis, there will be chaos.

In terms of the dynamics of the Unconscious and a hierarchical structure of the mind, I draw the conclusion here that – in spite of differences – we can see both in the Kleinian model (particularly in Segal’s and Bronstein’s development) and in Green’s model, a potential for compatibility with the economic model if we add to them the free energy model.

In that description, we can see the same process formulated by Green when he described the dynamics of the Unconscious and by Kleinians when they described the dynamics of Unconscious Phantasy. Therefore, I would argue that the Fristonian “hypothesis” can be seen as compatible both with Klein’s notion of unconscious phantasies and with Green’s understanding of the dynamics of the Unconscious.

Specifically: in Kleinian language, the “Fristonian hypothesis” describes Unconscious Phantasies including elements of constructing early (and even primal) unconscious phantasies, whereas in the Green model it would be equivalent to the representations cathected with affect, including the earliest ideational representations and the quota of affect.

## What neuroscience can offer in this respect: Friston’s free energy

Why did Freud fail in his attempt to find a source of free energy which gives impetus to drives and indeed to mental life? What was he seeking? His concept of drives was based on energy which demands work of the mind: “a drive appears to us as a concept on the frontier between the mental and the somatic, as the psychical representative of the stimuli originating from within the organism and reaching the mind, as a measure of the demand upon the mind for work” (Freud, 1915b, pp. 121–122). The crucial phrase here is *demand for work;* it could not be explained in Helmholtzian terms of free energy (which Freud tried to use) because Helmholtz’s formulation described energy which is available, but Freud needed a formula for energy which demands.

Only relatively recently, the work of G. Hinton and others has allowed the development of what we can call Friston (2010) free energy. This kind of “information energy” responds well to Freud’s hypothesis. It is energy which demands (a need for minimizing it), and it is energy which crosses the border between somatic and mental. That demand can be seen as a force for organizing a structure (anti-entropy) which is necessary feature of a living organism. It seems natural to seek an answer to the question: “what is the source of the drives’ energy?,” in the notion of free energy and predictive coding.

In recent years, the “Bayesian model of the brain” (Friston, 2008) which describes the brain as predictive agent and the “free energy principle” (Friston, 2010) have become very popular in theorizing on the interface between the brain and different aspects of the mind. These models are complex biophysical models described in mathematical language and it is not my goal here to provide an in-depth explanation. For the purpose of this paper, I need to restrict my explanation to underlining particular dimensions of these theories which I found relevant to the contemporary psychoanalytic theory of the drives. I also use the hypothesis presented by Fotopoulou and Tsakiris (2017) on the development of “the self,” in which they use the “free energy principle” as the first and very convincing example of how it can be understood as a model of the somato-psychic frontier. I will claim that their work, based mainly on empirical research, describes from a new perspective phenomena which were observed by psychoanalysts.

The Bayesian model, which can be extended beyond the human brain and may be used to describe a key feature of any living entity with the ability to react to the environment (Friston, 2013), assumes that such an entity creates a hypothesis about the environment prior to perception. Such an ability is vital for survival and further development because it allows a living organism to be prepared for a changing and sometimes hostile environment. On the most basic level, an organism can react accordingly to sensory input when it needs to (e.g., by moving away from a location if the temperature is too high and for that needs prior hypothesis of such situation). Of course, it is not possible that a hypothesis generated prior to perception would always match reality completely. What happens in such a situation? One possible reaction is an action to change the environment, whereas another is to modify the hypothesis. Friston (2010) explains the mechanism of this process using the Helmholtzian theory of free energy. “Free energy” in this model is a measure of surprise; in simple words, it is a measure of how much the hypothesis does not match reality. Free energy must be bound and can be bound either by modification of the hypothesis or by “action.” Both of these reduce the gap between hypothesis and reality. It is a property of this energy which creates demands on a living organism. At a biological level, it is a mechanism which maintains homeostasis.

I placed action in quotes because it may be actual action which leads to changes in reality, but also in more complex situations contains ambiguity and different possibilities; illusion psychologically speaking, may also be an action of the mind (Brown and Friston, 2012). I have chosen not to distinguish actual action and illusion here because they both minimize free energy, so for the free energy principle they both serve the same function. However, in my proposition of the drive theory they can serve as opposite forces.

Fotopoulou and Tsakiris (2017) adopted this model in an attempt to explain one of the earliest stages in human development. They formulated a hypothesis of the earliest development based on free energy and the predictive coding model. In the process which they call “mentalization^2^ “, the structure of a minimal self is developed. They write: “In development, as ongoing intersubjective bodily interactions with the caregiver get more complex, children build increasingly more sophisticated models of their own interoceptive states, as well as strategies for minimizing free energy in the interoceptive systems.” and “we have also argued that interoception, and in particular, the mentalization of interoceptive signals play a critical role in self-other boundaries. The distinction between self and other, which is crucial for self-awareness, *is equally essential* for awareness of other people,;” finally, they conclude: “Specifically, we have described as “embodied mentalization,” the process of building generative models by detecting regularities and irregularities in modality-specific and “amodal” properties, such as synchrony, and organizing sensory input of both personal and interpersonal origins into distinct, unitary multimodal schemata (perceptual inference). We have also stressed that such models refer not only to exteroception but also to interoception: the senses that inform the organism regarding the homeostatic state of the body. Furthermore, the mentalization of the body involves not only perceptual integration and subsequent inferences, but also action and thus sensorimotor integration (active inference). Accordingly, we have made the radical claim that in early infancy when the motor system is immature, proximal interactions are necessary for the active mentalization of interoceptive states and therefore the corresponding core aspects of the minimal self. There is therefore a continuity between the minimal and the interactive, social self.”

They claim that such a structure contains: (1) a representation of the infant’s own body including interoceptive, proprioceptive, and homeostatic signals; (2) a representation of a caring object; and (3) affect.

Now, I collate their account with Isaacs’s (1948) description of the process of forming earliest phantasies: “through a natural unity of rhythm between mother and child, or through the skillful handling of any difficulties that may arise, the infant is soon able to receive pleasurable satisfaction at the breast, good co-ordination of sucking; and a positive attitude to the sucking process is set up which goes on automatically thereafter, and fosters life and health. Changes of contact and temperature, the inrush of sound and light stimulation, etc. are manifestly felt as painful. The inner stimuli of hunger, and desire for contact with the mother’s body are painful, too. But sensations of warmth, the desired contact, satisfaction in sucking, freedom from outer stimuli, etc. soon bring about the actual experience of pleasurable sensation. At first, the whole weight of wish and phantasy is born by sensation and affect.”

I argue here that unconscious phantasies can be translated to Friston’s “prior hypotheses” (inference in Fotopoulou and Tsakiris’ paper), and a situation in which any hypothesis differs from the experience is producing free energy which has to be bound. It can potentially be seen as a source of energy which is needed to build or to modify the content of unconscious phantasies. Fotopoulou and Tsakiris’ description of the development of the minimal self can be seen as development of primal unconscious phantasies.

## My somato-psychic frontier hypothesis

In my hypothesis presented here, I assume that the qualitative aspect of the drive in the previously presented psychoanalytic schools and Freud’s original take, even if it differs in formulations, is described in compatible forms. There is an agreement that the drives’ aims and objects vary and change over time in the developmental process. The drives are unconscious; conscious or unconscious [repressed] affect give them impetus, and they invisibly steer human perceptions and actions toward human satisfaction.

I claim here that whether in the form of unconscious phantasy, or a set of representations, they are equivalent to “prior hypotheses” in Fristonian free energy theory.

If Fotopoulou and Tsakiris (2017) are correct, I would extend their claim that the same mechanism should apply to all inputs; thus, all mental phenomena and experienced inputs can potentially become embodied/cathected; and affects (which are always part of those inputs) are vehicles for psychic energy. Also, I believe that this mechanism operates throughout the entire human life.

This is in line with the Kleinian formulation of unconscious phantasies, which includes and emphasizes the significance of the relation between representations of the object and the self. In later development, this structure becomes more complex because prior cathexes influence perception, consequent actions, and subsequent inputs. This process creates the circular situation that is described by both Segal and Green. The main difference in that respect between Kleinians and Green lies in the perspective of their descriptions; object-relation, and individual inner perspective respectively. Again, what remains unknown in these formulations is what factor decides what and how something becomes cathected or not during the lifespan. As we know, not every experience can be corrective in terms of inner life.

In the Friston (2013) model, any living organism creates a hypothesis about the environment starting from very rudimentary data such as, for example, homeostasis; and any new change in a given environment releases free energy. During the earliest stages of human development, it must include mostly signals from the body: interoceptive, visceroceptive, and homeostatic states, but also elementary external inputs from objects or rather part-objects like sounds, touch. The dominance of bodily impulses in this stage explains the fact that the earliest hypotheses and mechanisms reflect bodily states; they constitute the bodily ego. I believe this process starts in the prenatal stage and the infant is born with some rudimentary hypotheses. Those prenatal hypotheses have been seen as innate or phylogenetic phantasies. They are heavily challenged after birth by a completely changed environment (both external and internal). Since development allows this possibility, it includes a richer spectrum of signals from the affective system and the external environment. I believe that Kristeva (1984, 2000) describes the same process from a different perspective. She believes that at first an infant experiences external input (including the mother) as sound, voice, rhythm etc. She calls it semiotic function and believes that together with bodily experiences and affect, they constitute “emotional experience, both psychical and subjective, based on the drives in an interpersonal context” (ibidem, 2000). This sounds very similar to Fotopoulou and Tsakiris (2017). Bronstein (2015) uses Kristeva’s work to illuminate the process of formatting the earliest unconscious phantasies and their role in mental functioning; she writes (ibidem, 2015) “In my opinion, these semiotic aspects that are part of the early relationships and phantasies of the mother’s body play a very important part in how we experience the world and communicate with others. Issues such as the patient’s rhythm of speech, the ‘musicality’ in their verbal expression, the intensity placed on their words, sensitivity to noises and to silences, and issues of space in relation to the analyst’s physicality can exercise a powerful effect in the session.” Both Kristeva and Bronstein believe these aspects of phantasy (thus drives) remain unconscious; Kristeva calls it the “pre-narrative envelope.” I add that the content of these phantasies (envelopes) is projected onto perception, emotional experiences, more mature aspects of mind like thinking and all other aspects of mental life.

I believe that the “envelope metaphor” applies well to all unconscious processes and phantasies; also, despite the fact that phantasies (and the whole Unconscious) operate as a whole hierarchical structure, there are also compartments within it; thus, we can say there are a number of envelopes there. The same applies to the mind as a whole, with the most important barrier being between the Conscious and the Unconscious. There are many notions of such barriers, starting from Freud’s “stimulus barrier,” Federn’s (1956) ego boundaries, and Bion’s (1962) “psychic membrane,” among many others. Rabeyron (2021) formulates an interesting hypothesis linking these psychoanalytic notions with the concept of Markov blankets, which is an important property of the Friston principle of free energy. In both psychoanalysis and Friston (2013) theory, the notion of a boundary between the outside and the inside of the system is crucial. It determines the ability to maintain an organically consistent system, but it also determines differentiation between the inside and outside environment. Furthermore, maintaining the organized system demands the ability to react to changes in the outside environment; this requires building hypotheses and reacting accordingly, but of course it also requires differentiation between the inner and outer system. Only then we do have a gap between hypothesis and reality which creates free energy.

From an analytic point of view, the most important barrier is between the inner world and external reality. The task of dealing with this is, according to Freud (1911), Ego function; and the process of adjusting to reality is called the “reality principle.” However, Freud (1911) writes:

“A general tendency of our mental apparatus, which can be traced to the economic principle of saving expenditure [of energy], seems to find expression in the tenacity with which we hold on to the sources of pleasure at our disposal, and in the difficulty with which we renounce them. With the introduction of the reality principle, one species of thought-activity was split off; it was kept free from reality-testing and remained subordinated to the pleasure principle alone. This activity is *phantasying*,”

I propose the following interpretation of this: saving energy, which for Freud is a basic principle, can be achieved in two ways. The first is through the “reality principle” which includes two elements: action to change reality or adjusting our goals and objects to reality (modification of our drives’ vicissitudes/hypothesis). The second way of saving energy can be creating an illusion [what Freud (1911) called hallucinatory wish fulfilment] that reality is exactly like our phantasy/drives’ vicissitudes/hypothesis. The latter serves the pleasure principle without the need to modify inner representations, but it needs to create a barrier which is the barrier between the Conscious and Unconscious. This barrier creates a “new frontier;” my understanding of this new frontier is the place where “the conflict between Ego (which represent reality) and Id” (Freud, 1920, 1923) takes place. Then we have another sub-system Super-Ego developed through cathexis and identification (Freud, 1923), which demands a new barrier. All new barriers are potential for conflicts and may create free energy, in Freud’s words: tension. Therefore, the Ego can mitigate potential conflicts between different agencies of the mind but also through defense mechanisms and creating illusions can create new frontiers which generate conflicts and potentially aggravate discrepancy between hypothesis/phantasies and reality.

As the body and the environment are dynamic structures, the organism (mind and body) is exposed to a certain amount of free energy most of the time, despite all efforts to minimize it.

Furthermore, I suggest that with growing complexity the initial conflict between reality and internal hypotheses/unconscious phantasies is supplemented by conflicts between different agencies of the mind; later by split-off or dissociated parts of the mind etc. (all these new sub-systems have to produce their own hypotheses at the same time as being hypotheses themselves). All this constitutes the conflictual character of the mind.

I also suggest that this process involves defense mechanisms which in this context can be described as agents of misrepresentation or creating illusion, which is to minimize free energy. From a psychoanalytic point of view, illusion is necessary to maintain the mind intact by negotiating internal conflicts, but can also be a cause of pathology when illusion takes over the reality principle (Steiner, 2020). From an energy-economic point of view (at least in Friston’s notion of it), an illusion can minimize free energy. Brown and Friston (2012) argue that “illusory percepts are, in fact, Bayes-optimal, and represent the most likely explanation for ambiguous input.” In other words, in a complex and ambiguous situation, the mind can produce an illusory explanation which can minimize free energy but falsely match a hypothesis with reality. Later, it has the potential to become part of another layer of internal hypotheses or, in analytic terms, to be cathected. Complexity and ambiguity may be created not only from overwhelming external input but also from conflicts between external input and already cathected representations of objects or wishes, and from internal conflicts between different psychic agencies or contradictory feelings. Those illusory ideas can also be cathected and become parts of unconscious phantasies. I suggest that such a process lays the ground for the Ego function that Lacan called “méconnaissance” (Lacan, 2014). During development, this function becomes one of the possible purely psychic “actions” alongside motor actions, which become behavioral and often serve as a means of communicating wishes to change, or adapt reality to the prior hypothesis. An example of a psychoanalytic description of such a situation may be any account of projective identification which may serve as only an illusion, but can also be actualized by forcing a real object to play a role prescribed in phantasy. A more complex account of the complexity of a relationship when a compromise between phantasy and reality of the object has to be found is in Joseph Sandler’s notion of role responsiveness (Sandler, 1976). The highest functional level of this process involving illusion is what Bion (1963) described as the K and –K dimension; where K stands for thoughts which represent reality (inner or outer) and –K stands for thoughts which represent a false narrative.

I believe that my hypothesis on drive energy can successfully complement contemporary psychoanalytic theory of drives and Friston’s theory of free energy in a similar way to how Fotopoulou and Tsakiris used it. In effect, it may constitute the model that adds to contemporary psychoanalytic views on drives an economic point of view containing a biological (in fact, biophysical) source of the drives’ energy. In summary, I hypothesize that the free energy postulated by Friston is the primal source of that energy. Furthermore, I argue that Fotopoulou and Tsakiris’ hypothesis can explain what energy, and in what way it is used to build the content of the qualitative dimension of the drives.

I postulate that the unknown “endo-somatic source of energy” may be Fristonian free energy, and the processes of cathexis and de-cathexis, as described by Green, can be seen as processes of minimizing (binding) free energy.

As mentioned above, the human mind is a very complex apparatus which contains subsystems and thus creates possible barriers/frontiers not only at the level of the biological body and environment, but also between purely psychical entities. This suggests that possible sources of free energy may have different locations. The free energy principle teaches us that free energy must be minimized by binding. This is achieved by two-way traffic. One direction is action: on a bodily level, for instance, it might be a release of hormones or any other somatic reaction; on the external level, it might be a motor reaction or illusion (including what Freud, 1900 described as hallucinatory wish fulfilment and negative hallucination). The other direction is the development of hypotheses. I prefer to say development instead of modification because, with time, a system of hypotheses becomes more and more complex. There are more data to deal with, while the ability to deal with such a growing magnitude of input is also growing. I suggest that those earliest hypotheses are what psychoanalysts call primary phantasies. They include bodily ego, primal objects, part-object, object-relations and so on. Different schools of psychoanalysis lay emphasis on different aspects of that process. I also suggest that the ability to develop those phantasies into a more mature form and to react adequately to a dynamic environment (internal and external) is the equivalent of what psychoanalysts call the Ego. The processes of modifying those internal hypotheses and actions to actualize them can be seen as the processes of de-cathexis and cathexis respectively. This happens on different levels of the mind, but the main “economic” mechanism remains the same.

Input in this process is the entire life experience; by life experience, I understand here a “product” created by perception and unconscious phantasies, which influence perception. This includes affect which is part of both perception and unconscious phantasy. Perception in this model may or may not be conscious. All this creates new hypotheses which make links between all experiences within a given moment. With maturation, those hypotheses should become more specific and selective; thus, hypotheses become more adequate. This process happens by building a hierarchical structure with more primitive and general content at the bottom, and more specific and complex content at the top. In psychoanalytic language, bottom stands for deep unconscious and top for conscious. However, this developmental process can be arrested by over-cathexis; a situation that one or more hypotheses/phantasies become dominant and rigidly defended against any modification. Such a defense can be carried out by means of illusion and/or behaviorally.

I believe this developmental process is the process that Kleinians call the development of unconscious phantasies. I suggest that the process of making hypotheses or unconscious phantasies more selective includes the creation of representations and linking them with affect and simultaneously developing defensive mechanisms. In this way, representations of objects, representation of self, ideas and defensive strategies, become cathected and charged with affect, constituting complex hypotheses about life, the world and oneself. However, those hypotheses have ingrained in them the demand to be confirmed and cannot be given up, but as a whole structure can be gradually modified.

Any change in such structures needs de-cathexis. In other words, adaptation to a changing world demands a constant balancing of cathexis and de-cathexis. I am in agreement here with both Green and Penot that de-cathexis plays a crucial part in the processes of any psychic development such as subjectivization or mourning. Their theorizing on this issue is in line with Britton’s (1998c) observation that mourning and development requires a relinquishment of believes (ideas or objects). However, I would go further and claim that de-cathexis has a part in every and any process of restructuring unconscious phantasies. In my understanding, it means changes to representations of objects, self, and so on.

However, it confronts us with an important paradox; on the bodily (biological) level, maintaining homeostasis is the main factor keeping an organism alive, so maintaining a low level of free energy is desirable. Although, in terms of “psychic life,” “Nirvana principle” (Freud, 1920), also called “inertia principle,” which is a tendency to keep tension/free energy constantly at low a level is an anti-life force (death drive), free energy and dynamics cathexis/decathexis/new cathexis is a condition of “*sine qua non*” for any psychic development.

In this model, the energy of Freudian drives in its “pure” form is “Fristonian” free energy. As described by Friston, two ways of binding energy make drives dual; I can call them simply forces of cathexis – de-cathexis. At the start of this process, this free energy is a biophysical phenomenon and an intrinsic feature of any living entity; also intrinsic is the necessity to minimize it in two ways simultaneously, as described above.

In the relatively long time which is needed to achieve a high level of complexity in human experience, they become not only dual but also dialectic in their character (Segal, 1993). The drives are necessary for creating the potential to develop the mind, but they can also restrain this potential.

Both sides of this dialectic pair are absolutely crucial. The dialectic character of the drives is essential because only these two forces operating together can support development; as Penot observed, the permanent dominance of either of the two can arrest development. We have many clinical accounts of pathology coming from such arrests. For example, Bell (2015) tries to classify phenomenologically some of the clinical expressions of such a situation under the aegis of the death drive. In such a context, I would suggest that what he calls the death drive is either the dominance of cathexis in the form of living in phantasy (trying to impose it on the external world or subject is isolating from the world), or dominance of decathexis in the form of a chaotic, fragmented and split mental life. Feldman’s (1997) account of projective identification in the session is, in my view, captured exactly in this line: “From the patient’s point of view, the projections represent an attempt to reduce the discrepancy between the phantasy of some archaic object relationship, and what the patient experiences in the analytic situation.” I think it is a situation when the patient’s psychic apparatus cannot modify its hypothesis and tries to impose hypothesis on reality, in the framework of my proposition it is a situation when the balance between drives is distorted toward cathexis. I also fully agree with Bell in his clinical observation and argument, however, I would suggest that on a metapsychological level what he calls death drive is a severe imbalance between two dialectical forces, rather than the “death drive” as a sole force.

I emphasize their dialectic character, proposing “balancing” instead of Green’s term “oscillation” (Green, 2015a), because they operate at the same time and have the same source of free energy. When observing the vicissitudes of the drives, I believe that what we see as fusion and de-fusion are in fact different configurations and stages in balancing between the two ways of minimizing free energy, which initially cannot be dual in its nature and achieves a dual character in the process of minimizing and transforming it into the drives’ energy. Another important factor in this development is that the human mind also uses “the third way” of minimizing free energy, which is illusion (which in a broad sense is the equivalent of deceiving one’s own consciousness); although we have to remember that the broad church of “illusion” includes some potentially pathological mechanisms such as massive projective identifications, but also defense mechanisms which can preserve the balance of the mind in excessively complex situations. Developmental mechanisms such as symbolization, which is crucial for mature functioning, also include elements of illusion (compare with Rabeyron, 2021) etc. To capture all these nuances will demand a separate body of work but in this paper I am proposing only the framework.

Another crucial factor is the time needed to achieve complexity in these internal hypotheses. This is due not only to the maturation of the brain but also, as clinical observations teach us, because changes in unconscious phantasies can be made only gradually. Perhaps too much surprise (free energy) is so overwhelming to the system that the balance between the two directions of the drives is disturbed to a degree that arrests development. I would suggest that this might be the biophysical equivalent of what Winnicott (1965) called impingement, and what lies behind the overexcitement described by theoreticians of the Paris School of Psychosomatics.

## Possible further directions for development

If my hypothesis is correct, it might open some new possibilities for work on metapsychology. In my view, there are at least five issues, as follows:

(1) We might consider the biggest developmental crises to be underpinned by significant changes in the internal and external environment, which lead to higher levels of free energy so that, subsequently, we observe a higher pressure in the drives. For example, this could explain the fact that adolescent crises are experienced at different ages by different persons and do not always occur at the same time as biological maturation (e.g., hormone activity).

Similarly, we can think about the psychoanalytic situation exactly in the way that Scarfone (2018) writes that psychoanalysis: “presents patients with new packets of free energy.”

(2) One of the controversial topics in psychoanalysis is the location of aggression in drive theory: i.e., the question of if or when aggression is self-preserving; if and when it is a derivative of the “death drive;” and so on. In my hypothesis, we can see aggression as an affective element of the unconscious phantasy/hypothesis, which could be considered to be part of a complex structure and not rigidly attributed to any of the drives. However, aggression as affect and as behavior can be linked to the “action” side of the dialectical pair of drives; thus, rather as a force of conservatism in Segal’s words. Nevertheless, this does not qualify aggression as a negative force.

Destructiveness can be linked with aggression or passivity alike but not directly and not always. I would suggest that qualifying a phantasy as destructive should depend on the role it plays in the dimension of adaptation/development vs. stagnation/maladaptation, rather than on descriptive features.

(3) Another concept arousing discussion is the infant’s envy, particularly in the earliest stages of human life. In the light of my proposition, envy seems to be a derivative of a way of minimizing free energy: an obvious consequence of the massive challenges of an environment which is changing fast and radically. The infant moves from the relatively stable environment of the womb to a much more changeable life. An infant is exposed to more and more new perceptions which cannot as yet have counterparts in internal hypotheses (e.g., the need to scream when a hunger is a new experience). The most crucial challenge seems to be a clash between experienced dependency and the omnipotent solitude of a baby in the womb. This kind of reaction (an envious wish to destroy) leading to the destruction of a reality that is too new seems to be inevitable. However, Winnicott’s (1955) claim concerning the role of the environment should be taken into account. I agree that the degree of this challenge (and thus the amount of free energy that drives envious reaction) can be mitigated or increased from outside; namely, by a mothering person.

(4) As Bronstein (2011) noted, one of the most important differences between approaches in psychosomatics is a different understanding of the role of drives and different models of drive theory. This new proposition may help discussion in the psychosomatic field. One example is the question of whether psychosomatic symptoms are part of unconscious phantasies, or a direct product of the death drive which blocks the development of psychic structure. Another example is the question of whether psychosomatic symptoms are caused by excessive drive energy, or rather by an imbalance of drives which arrested development on the somatic experience level, and prevented symbolization.

(5) Additionally, it might be possible in further theorizing to explain, in terms of mathematical logic, Bion’s (1963) concepts of containment and “grid” using Tarski’s (1933) undefinability theorem and his work on language and the definition of truth, which would help us to understand predictive coding in a relational context.

## Conclusion

Firstly, I need to conclude that despite the fact that we have various descriptions of a role and the dynamics of the derivatives of the drives, it is common to all psychoanalytic schools of thought that we cannot have access to the drives as such. Taking seriously the idea that all mental activities are underpinned and powered by drives, I find both Green’s take on the drives and the Kleinian notion of Unconscious Phantasies as representative of the drives very convincing. It comprehends all aspects of transformations and various forms of the drives’ vicissitudes. Bronstein’s (2015) contribution, which I see as a multi-layered hierarchical model, makes it much more compatible with other schools’ approaches, such as the contemporary Freudians’ focus on ego development (Sandler and Schachter, 2014), or those that focus on sensitivity to input from the environment (Winnicott, 1965). Segal’s (1994) contribution describes a particular function of unconscious phantasies as internal hypotheses; consequently, she maps out the nature of the interface between those internal hypotheses and reality.

This paper has argued that free energy as described by Friston can be seen as the source of the drives’ energy in psychoanalytic theory. Similarly, the minimizing of free energy can be seen as binding it; this process would be the Freudian “somato-psychic frontier.” In this model, tension can be understood as a high level of free energy created by a gap between reality and prior hypotheses/unconscious phantasies (also between different “departments” of the mind). A good example of this would be Bion’s (1979) “emotional storm,” which in his view is the unavoidable tension when two personalities, previously unknown to each other, meet. If cathected, needs, impulses, instincts (in the biological sense), and ideas become parts of vicissitudes of drives; the drives acquire aims and objects in that process, and are organized in the form of unconscious phantasies/hypotheses. Subsequently, we can understand the “pleasure principle” as a principle minimizing free energy; thus, the state of pleasure as a low level of free energy and displeasure as a high level of free energy. The “Reality Principle” would be necessary to achieve a high level of adequacy between unconscious phantasies and reality, which also minimizes free energy but in a different more adaptive way. The role of erotogenic zones can be understood as sources of new and early significant impulses cathected and often linked with positive affect; in this way, they are incorporated into unconscious phantasies and achieve dominant status at a given time, but might be modified later by further decathexis and cathexis. I see this proposition as a complement to the current psychoanalytic theory of the drives; in particular, Kleinian and Green’s understanding of the drives. It might make the economic dimension of Freudian theory an important part of psychoanalytic metapsychology again.

## Data availability statement

The original contributions presented in this study are included in the article/supplementary material, further inquiries can be directed to the corresponding author.

## Author contributions

The author confirms being the sole contributor of this work and has approved it for publication.

## Footnotes

1 As already stated, in English translations of the Freudian “Trieb,” sometimes the word “instinct” is used; and sometimes “drive.” This causes confusion; I use the word “drive” to clearly distinguish it from instinct, which in other translations has a double meaning.

2 By mentalization they understand “progressive process of build-up of multisensory predictions in perceptual inference”.
